# Influence of Obstructive Uropathy on Cyst Formation and Nephrogenesis: Insights from a Fetal Lamb Model

**DOI:** 10.3390/jdb14010005

**Published:** 2026-01-09

**Authors:** Kohei Kawaguchi, Takuya Kawaguchi, Juma Obayashi, Yasuji Seki, Kunihide Tanaka, Kei Ohyama, Junki Koike, Shigeyuki Furuta, Kevin C. Pringle, Hiroaki Kitagawa

**Affiliations:** 1 Department of Pediatric Surgery, St. Marianna University School of Medicine, 2-16-1 Sugao, Miyamae-ku, Kawasaki, Kanagawa 216-8511, Japan; k3kawaguchi@marianna-u.ac.jp (K.K.); t2kawaguchi@marianna-u.ac.jp (T.K.); skchldrnshp@gol.com (Y.S.); k3tanaka@marianna-u.ac.jp (K.T.); k2oyama@marianna-u.ac.jp (K.O.); its0408@marianna-u.ac.jp (S.F.); 2 Department of Pathology, St. Marianna University School of Medicine, 2-16-1 Sugao, Miyamae-ku, Kawasaki 216-8511, Japan; j2koike@marianna-u.ac.jp; 3 Department of Obstetrics and Gynecology, University of Otago, Wellington, 23A Mein Street, Newtown, Wellington 6021, New Zealand; kevin.pringle@otago.ac.nz

**Keywords:** fetal cystic dysplastic kidneys, β-catenin, CD10, fetal lamb, obstructive uropathy

## Abstract

Obstructive uropathy (OU) during fetal development induces a fetal cystic dysplastic kidney. The mechanisms of cyst formation and the onset of renal dysfunction remain unclear. Determining whether nephrogenic potential persists during fetal life may suggest whether early intervention could preserve renal development. We aimed to evaluate residual nephrogenic activity in fetal cystic dysplastic kidneys using β-catenin and CD10 immunostaining, and to assess whether the site of obstruction influences cystogenesis. After appropriate approval, 20 timed-gestation fetal lambs had OU created at 60 days. Males underwent urethral and urachal ligation (*n* = 8, 3 lost), and females underwent unilateral ureteric ligation (*n* = 8, 1 lost). Fetuses were sacrificed at 80 days (*n* = 6) and 140 days (term, *n* = 10), comparing kidneys with normal controls of the same gestational age using immunohistochemical staining for β-catenin and CD10. Developing fetal cystic dysplastic kidneys were identified at 80 days. β-catenin staining showed the absence of granular cytoplasmic expression in cystic regions, indicating arrested nephrogenesis. In male models, cysts originated exclusively from proximal tubules. Female models exhibited mixed proximal and distal tubular involvement. CD10 staining confirmed the loss of proximal tubular markers. Renal development remained arrested at term. Cyst formation disrupts renal development early in gestation, which persists until term. Differences in cystogenesis between the models suggest that the site of obstruction influences pathogenic mechanisms.

## 1. Introduction

Mammals develop three successive kidney structures during fetal life: the pronephros, mesonephros, and metanephros [1]. The first two regress in utero, while the metanephros becomes the permanent kidney. Normal kidney development requires the outgrowth of the ureteric bud from the mesonephric duct into the metanephric mesenchyme, where reciprocal interactions induce nephron formation. In humans, the metanephros appears at approximately five weeks of gestation [2]. Potter’s syndrome is well known [3], and although the original paper described bilateral renal agenesis, bilateral multicystic dysplastic kidney (MCDK) can also result in Potter’s syndrome. MCDK, a form of cystic dysplasia, is a dysplastic kidney primarily composed of multiple cysts [4]. Dysplastic kidneys are characterized by abnormal and incomplete differentiation of the metanephric mesenchyme and ureteric buds. Diagnosis is based on histological features, including fibromuscular tissue surrounding primitive collecting ducts. In MCDK, numerous large and small cysts are present, often interspersed with dysplastic tissue, including cartilage. These kidneys are essentially nonfunctional [5,6]. Obstructive uropathy (OU) induced early in nephrogenesis can result in MCDK. However, the precise mechanism of cyst formation remains unclear [7]. A fetal sheep model has been developed to study compensatory mechanisms in the contralateral kidney after unilateral ureteral obstruction [8]. Previous studies have indicated that the etiology and morphology of multicystic dysplastic kidney can vary depending on the site and timing of urinary tract obstruction, and that obstruction may interfere with nephron formation in the nephrogenic zone [4,9].

Clinically, multicystic dysplastic kidney (MCDK), a form of cystic dysplasia, is one of the most common congenital renal anomalies detected antenatally. While unilateral cases often involute spontaneously, bilateral involvement is incompatible with life, with most of these infants exhibiting Potter’s syndrome at birth. Understanding the mechanisms of cystogenesis is therefore critical for predicting prognosis and considering potential interventions.

Although previous studies have implicated signaling pathways such as Wnt/β-catenin [10,11] and transcription factors including PAX2 [12] in nephron differentiation, it remains unclear at which developmental stage obstructive uropathy irreversibly arrests nephrogenesis. Moreover, whether the site of obstruction determines distinct cystic phenotypes has not been systematically investigated.

The fetal lamb model provides a unique opportunity to study obstructive uropathy, as nephrogenesis in sheep closely parallels that in humans in both timing and morphology. Unlike rodent models, the larger size of the ovine fetus allows precise surgical manipulation and longitudinal evaluation of renal development, making it particularly suitable for translational research into fetal interventions.

Previous work from our group has also demonstrated that the pressure setting of vesico-amniotic shunts critically influences bladder and renal outcomes in fetal lambs with obstructive uropathy. In particular, low-pressure valved shunts (15–54 mmH_2_O) preserved bladder compliance and renal development, whereas high-pressure shunts (95–150 mmH_2_O) failed to provide adequate decompression and resulted in outcomes similar to non-shunted obstruction [13]. These findings highlight that both the timing of intervention and the mechanical characteristics of shunting devices are crucial determinants of fetal renal and bladder development. Building upon this knowledge, the present study focuses on the intrinsic mechanisms of cyst formation in fetal cystic dysplastic kidneys, aiming to clarify whether nephrogenesis is arrested at specific developmental stages and how the site of obstruction modifies cystic phenotypes.

Elucidating the timing and mechanisms of cyst formation in fetal cystic dysplastic kidneys may inform strategies for early intervention, such as pressure-limited shunting, aimed at preserving nephrogenic potential. Identifying reliable molecular markers could also improve prenatal diagnosis and guide clinical decision-making.

In this study, we aimed to investigate the mechanism of cyst formation in ovine fetuses. Animal models, including fetal sheep, have been widely used to investigate the pathogenesis of urinary tract obstruction and its impact on renal development [4,9]. Previous studies have demonstrated that β-catenin plays a critical role in nephron progenitor cell differentiation and the mesenchymal-to-epithelial transition during kidney development [10]. We have also studied the presence of the paired box genes 2 (PAX2) and CD10 at 60 days of gestation in normal fetuses and in term OU kidneys in an ovine model [14]. This study aimed to evaluate residual nephrogenic activity in fetal cystic dysplastic kidneys using β-catenin and CD10 immunostaining, and to assess whether the site of obstruction influences cystogenesis.

Taken together, our previous findings on the influence of obstruction site, the role of β-catenin in nephrogenesis, and the expression of PAX2 and CD10 provided the rationale for the present study, which investigates whether nephrogenesis is arrested at specific developmental stages and how the obstruction site modifies cystic phenotypes.

## 2. Materials and Methods

### 2.1. Surgical Procedures (Ewe)

After approval was obtained from the Animal Ethics Committee of the University of Otago, Wellington, New Zealand [approval numbers AEC 1-16, AEC 3-18], timed gestation pregnant ewes were transported from the farm 24 to 72 h before the operation. Prior to transport, they were examined by ultrasound to confirm the pregnancy and to avoid unnecessary operations. Our perioperative and anesthetic management has been reported previously [13]. Ewes were fasted for 24 h with free access to water. Anesthesia was induced with nitrous oxide, oxygen, and halothane, followed by intubation with a cuffed endotracheal tube, and maintained with nitrous oxide/oxygen and 1–2% halothane, as described in earlier ovine models [15]. The term for a sheep fetus is 140–145 days.

### 2.2. Surgical Procedures (Fetus)

To investigate the effect of obstruction site on renal development, we established two obstructive uropathy models in 60-day-old fetal lambs, which is early in nephrogenesis of the metanephric kidney: urethral and urachal ligation in males and left ureteric ligation in females. This gestational age corresponds to early nephrogenesis, allowing assessment of the initial impact of obstruction. Silastic^®^ tubing (Dow Corning, Midland, MI, USA; 0.012 in, 0.3 mm diameter) was used for ligation, consistent with previous ovine studies [15]. This ligation method has been validated in previous ovine models and reliably induces obstructive uropathy. A total of 11 males and 9 females underwent surgery. These fetal surgeries were performed prior to our development of a reliable way to ligate the female urethra in fetal lambs of this gestational age [13]. For sacrifice, the ewes were anesthetized, and the fetuses were delivered by cesarean section.

### 2.3. Obtaining Renal Samples

The lambs were then euthanized using pentobarbital injected into the umbilical vein, as we have previously described [13]. 4 male lambs (2/4 fetuses survived) and 5 female lambs (4/5 fetuses survived) were delivered at 80 days, and the remainder at 140 days (term, 10/11 fetuses survived). Controls were normal fetal kidneys of the same period (3 fetuses at 80 days, 12 fetuses at 140 days). The lamb’s kidneys were then removed and fixed in 10% formalin for 24 h.

### 2.4. Histological Techniques

The kidneys were divided longitudinally, and samples were taken from the cut surface of the kidney, processed for light microscopy in paraffin blocks, and sliced at 2 μm for light microscopy. Histological sections were stained with hematoxylin and eosin (HE) to assess morphology. Immunohistochemical staining was performed to detect β-catenin (clone 14, mouse IgG1, BD Biosciences, Tokyo, Japan; product code 610154; antigen retrieval pH6; dilution 1:250) and CD10 (clone 56C6, mouse IgG1, Nichirei Biosciences, Tokyo, Japan; product code 413261; antigen retrieval pH9; used undiluted). CD10 was used as a proximal tubular marker [16]. Antigen retrieval was performed in citrate buffer (pH 6.0), followed by incubation with primary antibodies overnight at 4 °C. Secondary HRP-conjugated anti-mouse IgG was applied, and visualization was achieved using diaminobenzidine (DAB) with hematoxylin counterstaining.

### 2.5. Histological Assessment

Two or more pathologists blinded to group allocation examined the sections. β-catenin expression was classified as granular cytoplasmic (signaling) or linear intercellular (adhesion), and CD10 positivity was assessed in proximal tubular cysts. Morphological changes over time were assessed in HE sections. We attempted to use E-cadherin as a distal tubular marker; however, ovine-specific antibodies are not available, and human antibodies did not yield reliable staining in sheep tissue. Therefore, distal identification relied on morphology and the absence of CD10 positivity, which restricts definitive interpretation.

### 2.6. Analysis

Because of the small number of lambs available for this study, no statistical analysis was attempted. Only descriptive analysis was employed.

## 3. Results

### 3.1. Kidneys Available for Analysis

The kidneys from the urethral ligation model included 4 kidneys from 2 lambs at 80 days and 12 kidneys from 6 lambs at 140 days. For the ureteric ligation model, there were 4 kidneys from 4 lambs at 80 days and 3 kidneys from 4 lambs at 140 days (Table 1).

### 3.2. Cyst Formation

**Figure 1:**
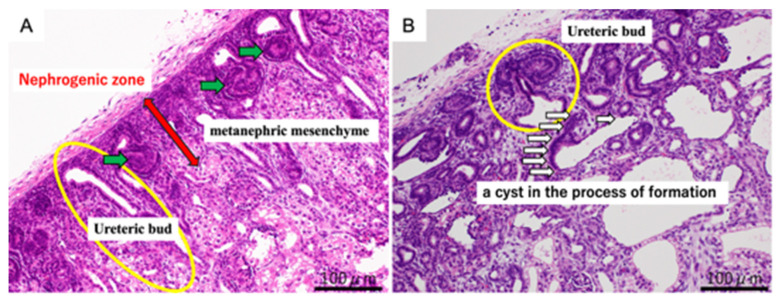
Representative hematoxylin and eosin (H&E) staining of fetal kidneys at 80 days of gestation. Panel (**A**) (normal kidney): Normal nephrogenic structures are highlighted. The red arrow indicates the nephrogenic zone, the green arrows point to the metanephric mesenchyme, and the yellow circle marks the ureteric bud. Panel (**B**) (obstructed kidney, OU): Developing cystic dysplasia is shown. The yellow circle indicates the ureteric bud, and the white arrows point to a cyst in the process of formation. Scale bar = 100 μm.

In both the urethral ligation and ureteral ligation models at 80 days of gestation, cysts were observed in the process of formation, accompanied by dilatation of the ureteric buds as well as the proximal and distal tubules (Figure 1). At 140 days of gestation, fully developed cysts were present in both models. These findings indicate that cyst formation begins before nephrogenesis is complete. The difference between models suggests that the anatomical site of obstruction influences tubular development and the pattern of tubular involvement.

### 3.3. Immunochemistry Results (β-Catenin)

#### 3.3.1. 80 Days

**Figure 2:**
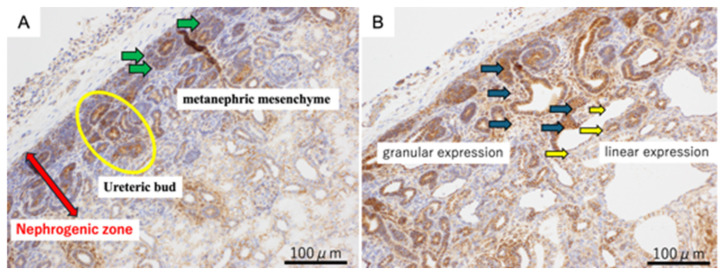
β-catenin immunohistochemistry of fetal kidneys at 80 days of gestation. Panel (**A**) (Normal kidney): β-catenin expression in normal nephrogenic structures. The red arrow indicates the nephrogenic zone, the green arrows point to the metanephric mesenchyme, and the yellow circle marks the ureteric bud. Panel (**B**) (80-day ureteric obstruction): β-catenin expression in the obstructed kidney showing cystic dysplasia. Dark green arrows indicate regions of granular β-catenin expression, typically seen in dysplastic nephrogenic zones. Yellow arrows highlight linear β-catenin expression along the epithelial lining of developing cystic structures. Scale bar = 100 μm.

In the 80-day ureteric obstruction kidneys, there were some areas of granular expression of β-catenin in the cytoplasm and some areas of intercellular linear expression between epithelial cells adherent to each other (Figure 2). In both the urethral ligation model and the ureteral ligation model, intercellular linear expression and cytoplasmic granular expression were observed in the ureteric bud, but only intercellular expression of β-catenin was seen in the cysts.

#### 3.3.2. Term

**Figure 3:**
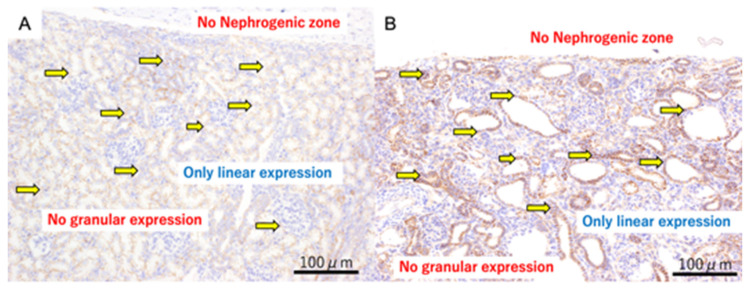
β-catenin immunohistochemistry of fetal kidneys at 140 days of gestation. Panel (**A**) (Normal kidney): β-catenin staining shows only linear intercellular expression; no granular cytoplasmic expression is observed. The yellow arrows indicate linear expression. Panel (**B**) (Ureteric obstruction): β-catenin staining also shows only linear intercellular expression; granular cytoplasmic expression is absent. The yellow arrows indicate linear expression. Scale bar = 100 µm.

At 140 days of gestation, cysts were well-established in both models. β-catenin positive staining was found only as intercellular expression and not in the cytoplasm (Figure 3).

### 3.4. Immunochemistry Results (CD10)

**Figure 4:**
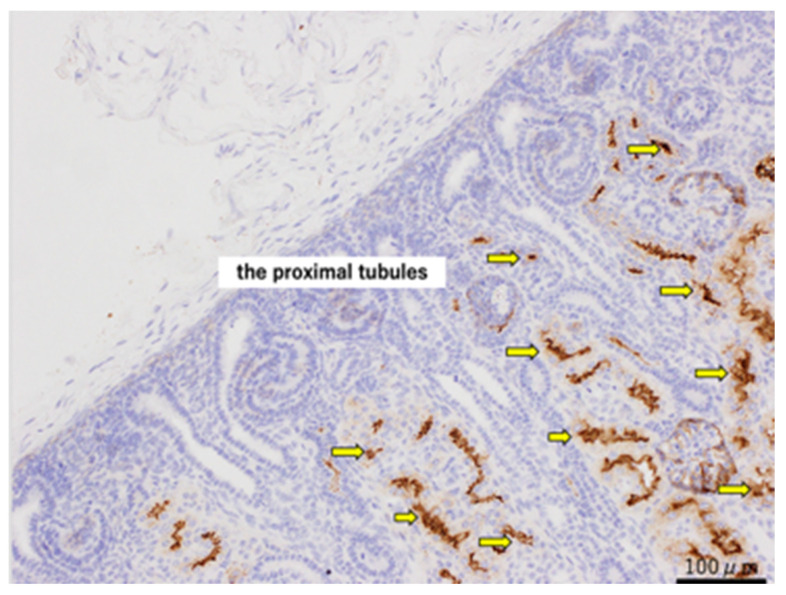
CD10 staining of normal kidneys at 80 days of gestation, shown at ×200 magnification. The yellow arrows indicate the proximal tubules. Scale bar = 100 µm.

**Figure 5:**
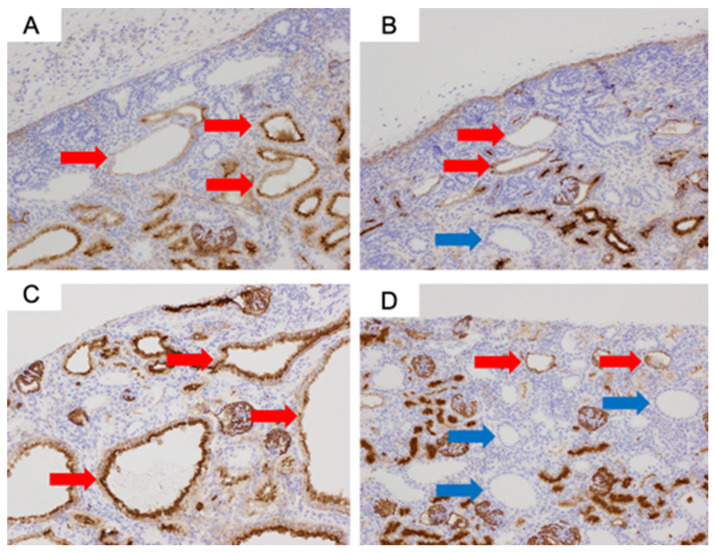
CD10 immunostaining of fetal kidneys at ×200 magnification: (**A**) Urethral ligation model at 80 days. (**B**) Ureteric ligation model at 80 days. (**C**) Urethral ligation model at 140 days. (**D**) Ureteric ligation model at 140 days. Red arrows indicate proximal tubules, and blue arrows indicate likely distal tubular cysts.

For the CD10 staining, CD10-positive cells were observed in the proximal tubules of normal kidneys at 80 days (Figure 4). In both the urethral ligation and the ureteric ligation models at 80 days, there were both CD10-positive and CD10-negative cysts. At 140 days, CD10-stained positive proximal tubular dilated cysts were observed in the urethral ligation model, but a mixture of proximal tubular and likely distal tubular cysts was observed in the ureteric ligation model (Figure 5).

## 4. Discussion

The pathogenesis of cystic dysplasia is still unknown, but various mechanisms have been considered [17]. Obstructive uropathy is one etiology that can possibly be treated during the fetal period. Established cystic dysplasia is not treatable. Therefore, in an attempt to assess whether it is possible to preserve renal function by early relief of OU, we created an OU model and explored the role of β-catenin and CD10. We hypothesized that the ability to mature and differentiate remains in the cystic dysplasia lesions, and we explored this association using β-catenin staining in this study. β-catenin has two functions. One is that it acts as an adhesion factor between cells in a mature individual. In this role, it functions as a downstream factor of E-cadherin. On immunohistochemical staining, this appears as linear positivity between the cells—intercellular staining. The other function is as a signal transduction factor represented by the Wnt-β-catenin pathway. Like E-cadherin, it is one of the many factors in this pathway [11]. The expression of β-catenin on immunostaining in embryonic kidneys can be seen as linear expression between cells and granular expression in the cytoplasm. Linear expression indicates an adhesion factor, and granular expression indicates a transfer factor. At the site of cyst formation, linear expressions were observed, but granular expressions were not observed. Thus, it was considered that the mechanism as a transfer factor was lost at the cyst formation site. β-catenin is essential for the mesenchymal-to-epithelial transition (MET), which is the critical developmental step during which nephron progenitor cells commit to epithelial differentiation. Without cytoplasmic (signaling) β-catenin, MET cannot be completed, and nephron progenitors fail to mature into functional tubular structures. Therefore, the absence of granular cytoplasmic β-catenin in cystic areas in our model indicates that nephrogenesis was arrested before the completion of MET. This provides a mechanistic explanation for why immature tubules dilate and form cysts following urinary tract obstruction. However, granular expression remains in the areas where there is dilatation of tubules which have not yet developed into cysts, suggesting that relief of obstruction, particularly through the use of vesico-amniotic shunting, at this stage may prevent the arrest of renal development. In the male specimens (urethral ligation), where only proximal tubule expression was noted, cysts appeared prior to the connection between the ureteric bud and the metanephric mesenchyme. In contrast, in the female specimens (ureteric ligation), which showed mixed expression, cyst formation seems to have occurred after this connection. These findings suggest that the mechanisms underlying cyst formation may vary depending on the developmental timing. We consider this an important point for future investigation.

The cause of MCDK has been studied for a long time, but there is still no definitive evidence as to the exact cause. Two theories have been proposed. One is the Bud Theory proposed in 1981 by Schwarz RD, Stephens FD et al. [18,19,20]. They hypothesized that ectopic ureteric buds may cause poor contact with the metanephric mesenchyme, which may result in abnormal metanephric development. In other words, it is a theory that abnormal ureteric buds fail to interact normally with the metanephric mass, resulting in immature nephrons. Other researchers have supported this concept [21,22]. In contrast, Matsell et al. reported that molecular markers expressed in the mature kidney were found in fetal MCDK and claimed that normal nephrogenesis occurred prior to the onset of renal dysplasia [12]. Our results indicate that cysts in cystic dysplasia arise from immature tubules and generally fail to mature. However, our previous studies have clearly demonstrated that appropriately timed vesico-amniotic shunting can allow developing cysts to resolve and restore normal histology [13,15,23]. In particular, Kitagawa et al [23] Kitagawa et al. (2003) showed that if the bladder is shunted too late, the cysts fail to resolve, underscoring the importance of optimal timing. These findings suggest that there may be a therapeutic window during which relief of obstruction can preserve nephrogenesis. Thus, while cyst formation typically represents arrested development, timely decompression of the urinary tract may prevent progression to nonfunctional dysplasia. In 2015, Qiusha Guo et al. reported that the calcineurin-NFAT signaling pathway is related to renal development and MCDK formation [24]. In addition, there are NPHP3/Nphp3, SIX1, and PAX2 genes that are reported to be involved in MCDK. It has also been reported that mutations in the HNF1β gene are strongly associated with the onset of MCDK [17,25,26,27]. Genetic analysis is a limitation of this study because we used sheep fetuses, and the genomic analysis of sheep has not yet been elucidated.

Our findings over the years suggest that cyst formation in cystic dysplasia begins early in fetal life, possibly as a result of obstruction during nephrogenesis, and remains arrested until term, unless the obstruction is relieved. While this may inform future research into potential prenatal interventions, our study was not designed to evaluate therapeutic strategies, and caution is warranted in extrapolating these results to clinical practice. In this study, different obstruction sites were used in male and female fetuses. At the time of the experiments, reliable urethral ligation in female fetal lambs was technically difficult, which necessitated the use of unilateral ureteric ligation in females. This methodological constraint introduced variation in the anatomical site of obstruction, which may influence cystogenesis. Although sex and obstruction site could theoretically confound interpretation, our previous ovine models of obstructive uropathy have consistently shown no significant histological differences between male and female fetuses, and in fact, we have never observed such differences in any of our OU models. Therefore, the observed differences in cystic phenotypes are most likely attributable to the site of obstruction rather than to biological sex. This represents an important limitation of the study that should be considered when interpreting the findings [14,15,28,29]. Clinically, potential interventions to preserve nephrogenesis in obstructive uropathy include vesico-amniotic shunting, pressure-limited shunts, and fetal endoscopic procedures. In the ovine model, nephrogenesis begins at ~60 days of gestation, corresponding to ~10 weeks in humans, and term at 140–145 days parallels human term at 40 weeks. Thus, the timing of intervention in sheep provides translational insight into the human fetal window for preserving renal development.

Future studies should explore the immunohistochemical changes associated with timely decompression of the urinary tract. Our previous work has already demonstrated that appropriately timed vesico-amniotic shunting restores normal histology in the ovine model [23]; however, the molecular and immunohistochemical correlates of this finding remain to be investigated. In addition, adjunctive therapies targeting signaling pathways such as Wnt/β-catenin or transcription factors like PAX2 could further enhance renal development. Genetic analyses, including evaluation of HNF1β and other candidate genes, may also clarify the molecular mechanisms underlying cystogenesis. Ultimately, integrating molecular diagnostics with fetal intervention strategies could provide a translational pathway toward improving outcomes in severe obstructive uropathy and preventing progression to nonfunctional cystic dysplasia.

Limitations: This study has several limitations. First, distal tubular identification relied on morphology and CD10 negativity. Because ovine-specific antibodies are not available and human antibodies did not yield reliable staining for markers such as E-cadherin, distal assignment remains tentative. Second, only β-catenin and CD10 were assessed as markers, which provides a limited view of nephrogenesis; inclusion of additional markers could yield a more comprehensive understanding. Third, the study was primarily qualitative and observational. The relatively small sample size and the inherent constraints of the ovine fetal model precluded robust statistical analyses. Fourth, interpretation of immunohistochemical staining patterns should be made with caution, as expression does not directly equate to functional outcomes. Finally, because reliable urethral ligation in female fetal lambs was technically difficult, unilateral ureteric ligation was used in females. This methodological constraint introduced variation in the anatomical site of obstruction, which could confound sex-related comparisons. However, our previous ovine models of obstructive uropathy have consistently shown no significant histological differences between male and female fetuses, suggesting that the observed differences are more likely attributable to the site of obstruction rather than biological sex. Thus, the results should be considered descriptive, and future studies with larger cohorts, expanded marker panels, and complementary functional analyses are needed to provide quantitative validation.

## 5. Conclusions

The expression pattern of β-catenin in the OU model suggests that urinary tract obstruction may prevent normal kidney development. In the fetal lamb model, it is necessary to treat before 80 days. Moreover, the pattern of cyst formation differs depending on the occlusion site.

## Figures and Tables

**Table 1:** Distribution of samples across groups and time points.

Group/Intervention	Time Point (Days of Gestation)	Number of Kidneys	Number of Fetuses
Urethral ligation (male)	80 days	4	2
Urethral ligation (male)	140 days (term)	12	6
Ureteric ligation (female)	80 days	4	4
Ureteric ligation (female)	140 days (term)	3	4
Control	80 days	3	3
Control	140 days (term)	12	12

## Data Availability

The raw data supporting the conclusions of this article will be made available by the authors upon request.

## Abbreviations

HE	Hematoxylin–eosin
MET	Mesenchymal–epithelial transition
OU	Obstructive uropathy
